# Role of magnetic resonance imaging in the management of perianal Crohn’s disease

**DOI:** 10.1007/s13244-017-0579-9

**Published:** 2017-11-15

**Authors:** Jose C. Gallego, Ana Echarri

**Affiliations:** 1 Department of Radiology, Complexo Hospitalario Universitario de Ferrol, Av. da Residencia s/n, 15405 Ferrol, Spain; 2 Department of Gastroenterology, Complexo Hospitalario Universitario de Ferrol, Ferrol, Spain

**Keywords:** Crohn’s disease, Magnetic resonance imaging, Perianal disorders, Diffusion magnetic resonance, Perfusion imaging

## Abstract

**Abstract:**

Perianal fistulas are a major problem in many patients with Crohn’s disease. These are usually complex fistulas that adversely affect patients’ quality of life, and their clinical management is difficult. Medical treatment sometimes achieves cessation of discharge and closure of the external opening; however, it is difficult to assess the status of the rest of the fistula tract. Magnetic resonance imaging is the method of choice with which to evaluate the condition of perianal fistulas and allows for assessment of the status of inaccessible areas. Magnetic resonance imaging also allows the clinician to evaluate other perianal manifestations of Crohn’s disease that differ from the fistulas. This imaging technique is therefore a fundamental means of patient monitoring. When used in conjunction with assessment of the patient’s morphological findings, it provides information that allows for both quantification of disease severity and evaluation of the response to treatment. New types of magnetic resonance sequences are emerging, such as diffusion, perfusion, and magnetisation transfer. These sequences may serve as biomarkers because they provide information reflecting the changes taking place at the molecular level. This will help to shape a new scenario in the early assessment of the response to treatments such as anti-tumour necrosis factor drugs.

***Teaching points*:**

• *MRI is the method of choice with which to evaluate perianal fistulas.*

• *In perianal Crohn’s disease, MRI is a fundamental means of patient monitoring.*

• *The usefulness of the Van Assche score for patient monitoring remains unclear.*

• *New MRI sequences' diffusion, perfusion, and magnetisation transfer may serve as biomarkers.*

## Introduction

Perianal fistulas are frequent manifestations of Crohn’s disease (CD) and cause significant morbidity, often with serious impairment of the patient’s abilities. Fistulas are abnormal communications formed by granulation tissue and are established between two epithelial surfaces. Perianal fistulas form between the inner surface of the anal canal (internal opening) and the skin (external opening). These fistulas are the main, but not the only, manifestation of perianal CD (PCD). Their treatment is difficult and sometimes requires colostomy or even proctectomy, although this does not resolve the problem in all patients.

Although CD-associated perianal fistulas only account for around 1% of all perianal fistulas [1], they appear in 30% to 50% of patients with CD and can be the first manifestation of CD as well as precede the finding of intestinal lesions in up to 30% of patients [2]. Additionally, the presence of such fistulas is associated with the most severe forms of CD [3].

Perianal fistulas seem to be caused by different pathogenetic mechanisms than enteric fistulas. In fact, up to 80% of patients with PCD do not have enteric fistulas [4]. Therefore, PCD is considered a different phenotype by the Montreal Classification, in which it appears as a differentiated subclassification [5]. It is possible that these lesions result from the deepening of distal rectal ulcers or fissures secondary to the forces exerted during defecation. It also seems clear that microbiological, immune, and genetic factors are involved in the aetiology of perianal fistulas [6].

Imaging techniques provide information on the anatomical and functional aspects of fistulas, which are often difficult to obtain through clinical examination, especially in patients with inflammation or abscesses. In addition, magnetic resonance imaging (MRI) makes it possible to evaluate the presence or absence of healing of the deep tissues of the fistula paths, a key aspect in the follow-up of patients.

## Imaging studies

MRI and ultrasound (US), both endoanal and transperineal, are imaging methods that show the anal anatomy in detail. They allow the clinician to assess the extent and complexity of disease as well as the presence of abscesses; such information is essential in choosing the most appropriate treatment. The higher anatomical resolution of these methods also contributes to successful surgical outcomes by decreasing the risk of incontinence or other complications. Unfortunately, this grade of precision cannot be achieved by other imaging methods such as fistulography or computed tomography.

Importantly, correct evaluation of patients with PCD should include an endoscopic study to evaluate luminal disease, specifically the condition of the rectum; a local imaging study, either MRI or US; and an examination under anaesthesia, during which abscesses can be drained, seton stitches can be placed in situ, and other necessary measures can be undertaken [7]. With respect to imaging, MRI is a more objective method than US, is more easily understood by nonradiologists, and allows for a more accurate comparison of the follow-up studies of each patient. In 2008, Sahni et al. [8] published a comprehensive review using methods of evidence-based medicine, i.e., consultation of guides from the American College of Radiologists and the American Gastroenterological Association, a review of the literature, and consultation with experts. The authors concluded that MRI is the best method with which to distinguish simple from complex fistulas, surpassing both endoanal US and physical examination. Conversely, a meta-analysis comparing MRI and endoanal US for detection of perianal fistulas concluded that the sensitivity of both methods is similar, although the specificity of MRI is substantially higher [9]. Transperineal US is also a very useful technique for the study of perianal fistulas. This technique is more comfortable and less invasive than endoanal sonography. However, although it was first described some years ago, it is only performed in a few centres. Bor et al. [10] stated that for patients with PCD, the accuracy of transperineal US is identical to that of MRI or endoanal sonography.

Several scientific societies and groups of experts are clearly committed to MRI for the study of PCD. The European Society of Crohn’s and Colitis (ECCO) [11] stated in 2010 regarding the diagnosis:

- 9A. *Pelvic MRI should be the initial procedure because it is accurate and non-invasive, although it is not needed routinely in simple fistulae [EL2b., RG B]*.
- 9C. *Anorectal ultrasound requires expertise, but can be equivalent to pelvic MRI in completing examination under anaesthesia if rectal stenosis has been excluded [EL5, RG D]. Fistulography is not recommended [EL3, RG C]*.

The ECCO-European Society of Gastrointestinal and Abdominal Radiology (ESGAR) Consensus [12] subsequently reaffirmed MRI as the most accurate imaging technique with which to study perianal fistulas, recognising that it also allows for the detection of many lesions that go unnoticed during an examination under anaesthesia and emphasising its role as a method for assessing the response to treatment:

- ECCO-ESGAR statement 5A*. MRI is the most accurate imaging test for perianal CD with accuracy surpassing examination under anaesthesia, and is recommended during the initial diagnosis unless there is a need for intermediate drainage of sepsis [EL 1]*.
- ECCO-ESGAR statement 5B*. Undetected or untreated fistulae extensions and abscesses are the major cause of treatment failure. Imaging, particularly using MRI, is highly accurate in detecting such complications and for treatment planning [EL 2]*.
- ECCO-ESGAR statement 5C*. MRI and endosonography are both superior to simple clinical evaluation at assessing treatment response, particularly for detecting residual abscesses, and either should be considered prior to significant changes in, or cessation of, surgical or medical therapy [EL 2]*.

The Shanghai Group, a group of experts comprising members of the World Gastroenterology Organisation, International Organisation for Inflammatory Bowel Diseases, European Society of Coloproctology, and Robarts Clinical Trials, stated in 2014 that US must be supplemented with MRI in many cases and that they consider MRI the gold standard imaging technique [13]:

Statement 8.1. *Pelvic MRI is a highly accurate non-invasive modality for the diagnosis and classification of perianal fistulas; therefore it is considered the gold standard imaging technique for perianal CD. MRI provides additional detailed information on luminal disease location, disease severity, and fluid collections*.

More detailed information on all recommendations of various consensus groups is provided in a review by de Groove et al. [14]. Notably, these authors stated that the use of MRI has reached a generalised consensus.

However, pelvic MRI also has its limitations. One of most important of its disadvantages is the difficulty that is often encountered in identifying the internal opening of the fistula. Other disadvantages include its high cost and contraindication in patients with pacemakers or other devices, claustrophobic patients, and patients with various other conditions.

## MRI findings

Generally, the correct identification of perianal fistulas is a complex process that requires an experienced radiologist [15]. The technical aspects of pelvic MRI when performed for evaluation of perianal fistulas have been widely published [16, 17]. It is important to obtain high-resolution images, tilt the oblique-axial and oblique-coronal planes according to the anal canal, and perform a T2-weighted sequence with a fat-suppression technique (e.g., in the axial plane) to enable easier identification of the fistula tracts and fluid collections. However, considering the advances during the last several years, two points could be modified: three-dimensional T2-weighted sequences could be performed instead of sagittal, oblique-axial, and oblique-coronal high-resolution T2-weighted sequences, and diffusion-weighted sequences could be added. The first change decreases the number of sequences and provides data for post-processing reformation of the images in any desired plane. The usefulness of diffusion-weighted sequences will be discussed later; however, we believe that no study protocols should lack the use of such sequences (Table 1).

**Table 1:** Possible protocols for MRI acquisition

	Sequence					FOV	Slice thick (mm)	Slice gap	Acc factor	Fat sat
	Philips	Siemens	GE	Toshiba	Hitachi					
Sagittal T2-weighted	TSE	TSE	FSE	FSE	FSE	260 × 260	≤4	0	2	NO
Oblique axial T2-weighted	TSE	TSE	FSE	FSE	FSE	260 × 260	≤4	20%	2	NO
Oblique axial T2- weighted with fat saturation	SPIR-TSE	FS-FSE	CS-FSE	MSOFT-FSE	FS-FSE	260 × 260	≤4	20%	2	YES
Oblique coronal T2-weighted	TSE	FSE	FSE	FSE	FSE	260 × 260	≤4	20%	2	NO
Oblique axial diffusion weighted imaging	Diffusion-weighted imaging EPI planar					380 × 380	≤4	10%	2	YES
Oblique axial 3D T1 W GE with fat saturation (Gd)	THRIVE	VIBE	LAVA	QUICK 3D	TIGRE	380X380	≤3	0	3	YES
3D T2 weighted sequence	VISTA	SPACE	CUBE	3D MVOX	isoFSE	260 × 260	≤1.5	0	3	NO

FSE, fast spin echo; TSE, turbo spin echo; SPIR-TSE, spectral saturation with inversion recovery turbo spin echo; FS-TSE, fat-saturated turbo spin echo; FS-FSE, fat saturated fast spin echo; THRIVE, T1-weighted high-resolution isotropic volume examination; VIBE, volumetric interpolated breath-hold examination; LAVA, liver acquisition with volume acceleration-extended volume; QUICK 3D, Toshiba name of the sequence, not an acronym; TIGRE, T1-weighted gradient echo with RF fat saturation; DWI, diffusion-weighted imaging; VISTA, volume isotropic turbo spin echo acquisition; SPACE, sampling perfection with application optimised contrasts using different flip angle evolution; CUBE, GE name of the sequence, not an acronym; 3D MVOX, 3D multivoxel; isoFSE, iso fast spin echo

Some additional findings of fistulas that are identifiable on MRI, such as the presence of abscesses, branching, and other features, are not included in other classifications. This led to the development of a radiological classification known as the St. James Hospital classification [18], which comprises five grades:

- Grade 1: Simple linear intersphincteric fistula. The tract runs between the skin and the anal canal, does not show branching, and does not surpass the outer sphincter or affect the ischioanal fossa.
- Grade 2: Intersphincteric fistula with abscess or secondary tract. Although complications occur, the disease never surpasses the outer sphincter. The branching or secondary tract can surpass the midline and show contralateral extension by adopting a “horseshoe” appearance.
- Grade 3: Trans-sphincteric fistula. The fistula tract passes through both sphincters and extends toward the skin through the ischioanal fossa.
- Grade 4: Trans-sphincteric fistula with abscesses or secondary tracts in the ischioanal fossa. The tract shows abscess formation, generally in the ischioanal fossa although sometimes also in the intersphincteric region, by adopting an “hourglass” shape.
- Grade 5: Fistulas that extend over the levator ani. Suprasphincteric fistulas run through the intersphincteric space to the highest point of the levator ani muscle, then pass through it, extending to the skin through the ischioanal fossa. Extrasphicteric fistulas originate from a pelvic organ, usually the rectum, and pass through the levator ani toward the skin, also through the ischioanal fossa. In any of these situations, contralateral extension may appear.

**Fig. 1:**
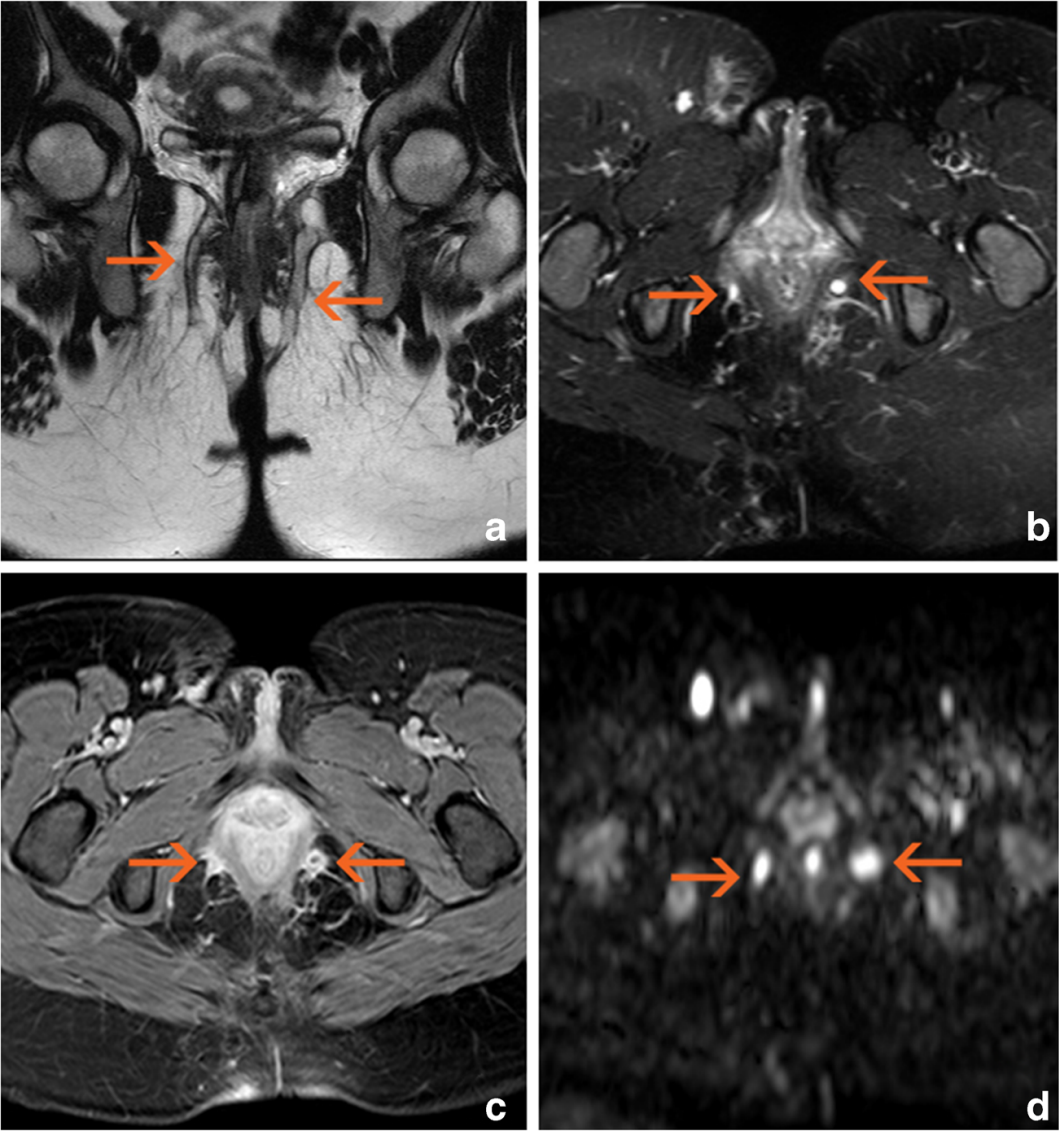
Complex bilateral transsphinteric fistula cursing across both ischioanal fossae (arrows). **a** Oblique-coronal T2-weighted image. **b** Oblique-transverse fat-supressed T2-weighted image. **c** Post-gadolinium oblique-transverse fat-supressed gradient echo T1-weighted image. **d** Native oblique-transverse native diffusion-weighted image with 800 s/mm^2^ b factor

**Fig. 2:**
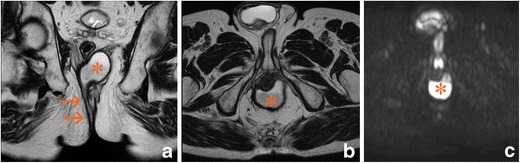
Right suprasphicteric fistula (arrows) with retroanal abscess (asterisk). **a** Oblique-coronal T2-weighted image. **b** Oblique-transverse T2-weighted image. **c** Native oblique-transverse native diffusion-weighted image with 800 s/mm^2^ b factor

Fistulas in patients with PCD are almost always “complex.” According to the American Gastroenterological Association [19], complex fistulas are defined as high fistulas, intersphincteric and trans-sphincteric fistulas, those that cross the levator ani muscle, and those with secondary tracts (Fig. 1). These characteristics increase the risk of complications. This is especially true for fistulas with secondary pathways; these tracts, when blind, can become complicated and lead to abscesses (Fig. 2). The most frequent complication is the presence of a branch coursing in the cranial direction from the highest point of a trans-sphincteric path, running toward the roof of the ischioanal fossa and even crossing the levator ani muscle. Contralateral tracts to the other ischioanal fossa, or “horseshoe” extensions, may also be present on both sides from the internal opening.

**Fig. 3:**
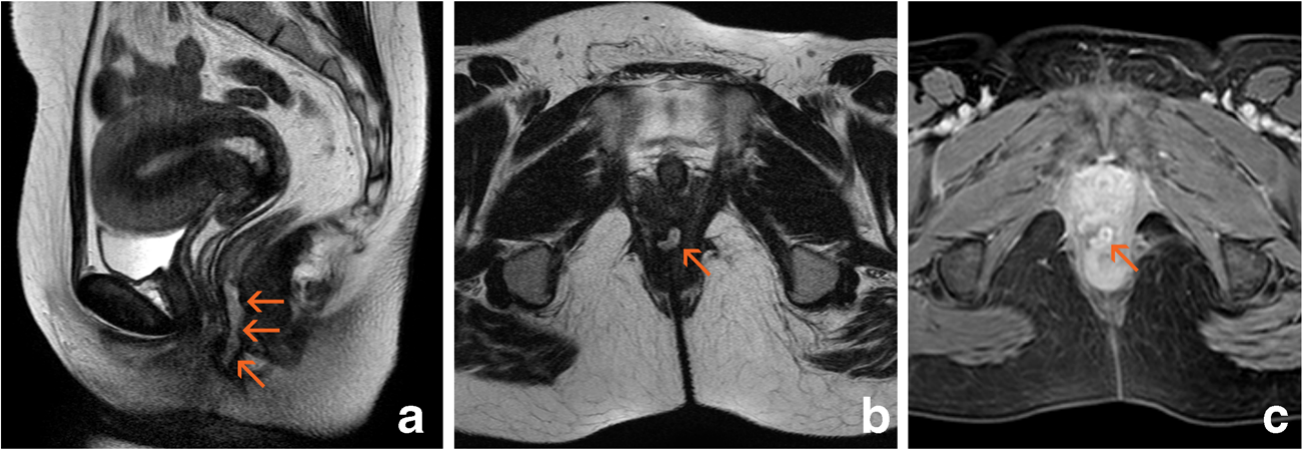
Anovaginal fistula. A thick gauge fistula exibited abscess formation involving the recto vaginal septum (arrows). **a** Sagittal T2-weighted image. **b** Oblique-transverse T2-weighted image. **c** Post-gadolinium oblique-transverse fat-supressed gradient echo T1-weighted image

Anovaginal fistulas (Fig. 3) also have special characteristics. Although they account for 10% of all fistulas in patients with PCD, they cause important problems associated with discomfort, infections, dyspareunia, and other conditions. They also have a worse prognosis because medical treatments are effective in only a low percentage of cases, the placement of seton stitches does not seem to be useful, and surgical treatment is associated with a high rate of recurrence [20]. In MRI, this may be the only indication for the use of endoanal coils because the pathways are better demonstrated given their smaller extension (2.0–2.5 cm) and the proximity to the coil [21]. However, the clinician must remember that other perianal fistulas may coexist; in such cases, it is mandatory to perform a second study with a conventional external coil.

Given the complexity of fistulas in patients with PCD, Horsthuis et al. [22] proposed a series of five steps to ensure an appropriate approach to pelvic MRI in patients with CD:

1. Identify each path and follow it throughout its course. If more than one path is present, it is very important to look for possible communications among them.
2. Look for abscesses and blind paths. They are more easily identified in T2-weighted sequences with fat saturation.
3. Check, preferably in the coronal sequence, if the pathways reach or surpass the levator ani muscle.
4. Identify the internal opening. It is usually located at the level of the dentate line (i.e., about 2 cm from the anocutaneous margin), although it can be located at any site.
5. Identify ancillary findings such as inflammation of other tissues (proctitis, infiltrates, bone oedema) or cancer.

**Fig. 4:**
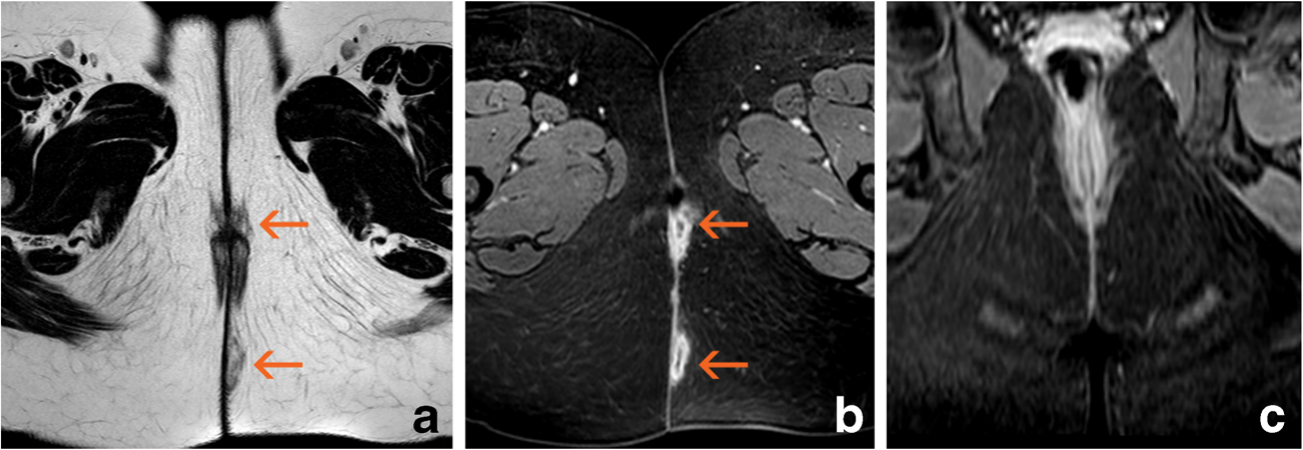
Hidradenitis suppurativa. Skin and subcutaneous abscesses (arrows) are present in the anal cleft without anal involvement. **a** Oblique-transverse T2-weighted image. **b** Post-gadolinium oblique-transverse fat-supressed gradient echo T1-weighted image. **c** Post-gadolinium oblique-coronal fat-supressed gradient echo T1-weighted image

Differential diagnoses should include pilonidal sinuses, haemorrhoids, and especially hidradenitis suppurativa, a disease that is associated with and may coexist with PCD, inducing clinical and histological confusion between the two diseases. The coexistence of abscesses in other locations such as the groin or axillae and the presence of multiple fistulas and blind pathways without a clear origin in the anorectal region suggest hidradenitis suppurativa. Pelvic MRI may reveal this disease if skin thickening and subcutaneous induration are present in the perianal area, anal cleft, and perineum (Fig. 4) [23, 24].

## Other possible lesions in patients with PCD

**Fig. 5:**
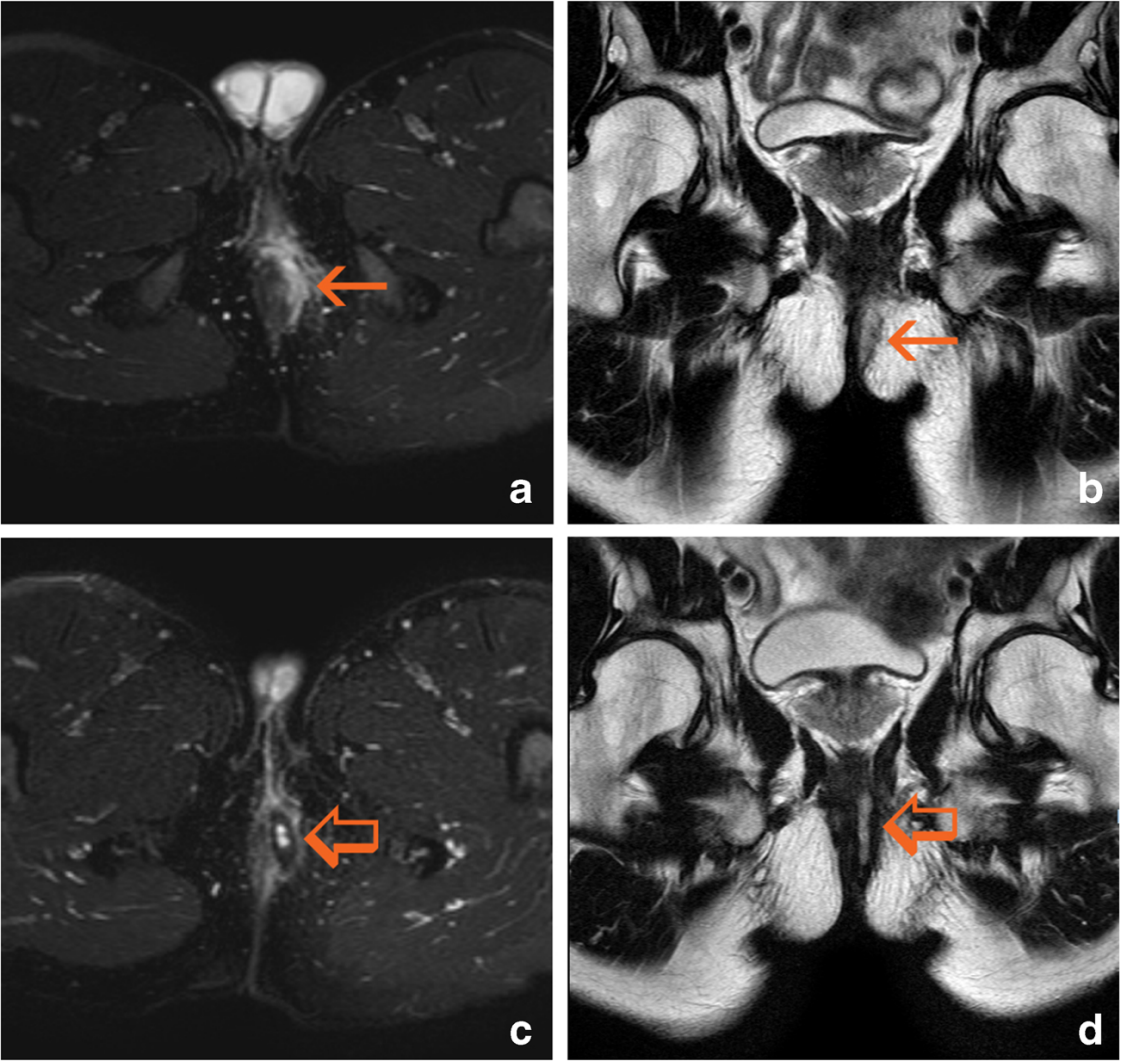
Anal ulcer. Diffuse hyperintensity of the left anal margin is present on T2-weighted images, and enhancement is present in the post-gadolinium sequence (arrows). A true fistula developed during follow-up (open arrows). **a** Oblique-transverse fat-supressed T2-weighted image. **b** Oblique-coronal T2-weighted image. **c** Oblique-transverse fat-supressed T2-weighted image. **d** Oblique-coronal T2-weighted image

**Fig. 6:**
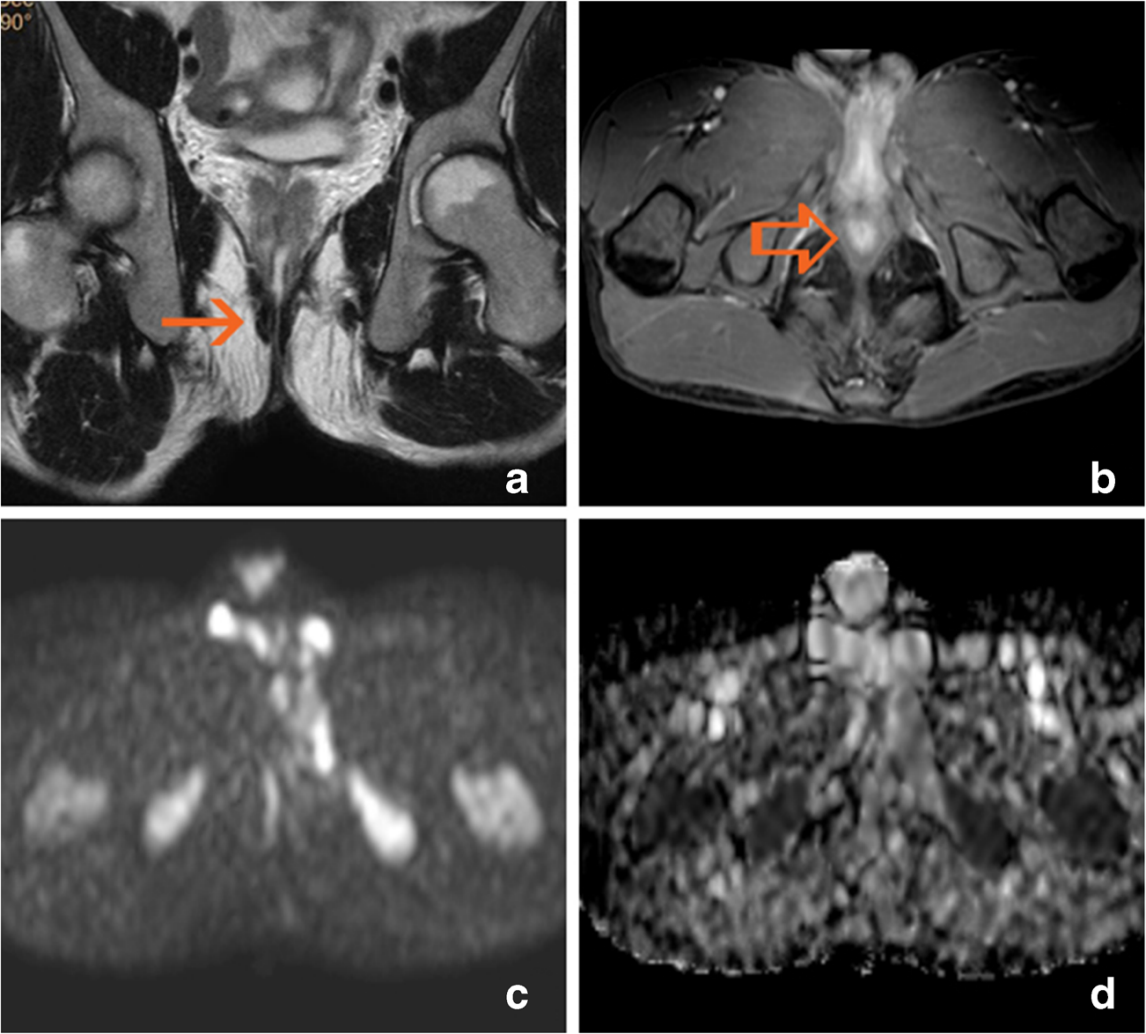
Anal fibrous stenosis. The internal anal sphincter shows hypointensity on T2-weighted sequences and diffuse enhancement after gadolinium administration (arrows); the native diffusion-weighted image and the ADC map show hypointensity. **a** Oblique-coronal T2-weighted image. **b** Post-gadolinium oblique-transverse fat-supressed gradient echo T1-weighted image. **c** Native oblique-transverse native diffusion-weighted image with 800 s/mm^2^ b factor. **d** ADC map

**Fig. 7:**
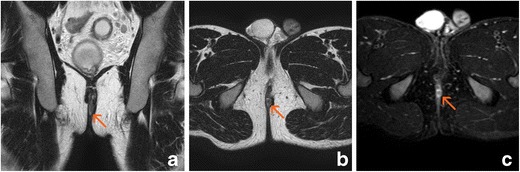
Cutaneous flap showing hyperintensity on T2-weighted images and a slight post-gadolinium rim enhancement (arrows). **a** Oblique-coronal T2-weighted image. **b** Oblique-transverse T2-weighted image. **c** Post-gadolinium oblique-transverse fat-supressed gradient echo T1-weighted image

In addition to fistulas, other less well-known manifestations of PCD also exist. Most result from the primary lesions caused by the disease [25]. Such manifestations include:

- Ulcerations: These lesions comprise both fissures and deeper cavitated ulcers. The latter can be very symptomatic and may result in the formation of a fistula (Fig. 5). When deep ulcers are present, there are almost always signs of proctitis. These ulcers are easily detected by MRI because they are usually associated with inflammatory infiltrates. They may be confused with infectious lesions, post-radiotherapy lesions, or ulcerated cancers.
- Stenosis: These lesions may be either inflammatory stenoses caused by anal spasm (type I) or true fibrous scar tissue (type II). They are usually asymptomatic until they reach a high degree of severity. A typical appearance of type II has been described as perianal hypointensity on T2-weighted images and peripheral anal enhancement after gadolinium administration [25] (Fig. 6).
- Cutaneous flaps: The cause of these flaps is lymphedema secondary to lymphatic obstruction, and 30% of flaps contain non-caseiform granulomas. They are usually located near the margins of superficial fissures and are almost always asymptomatic. Two types of cutaneous flaps exist. The first type of flap is large, oedematous, and cyanotic and typically appears alongside a healed ulcer. The second is called an “elephant ear” flap and is flat, soft, and painless. The flaps are usually hyperintense on T2-weighted images and show poor enhancement after gadolinium administration (Fig. 7).
- Neoplastic lesions: Patients with highly evolved perianal disease may develop malignant lesions such as anal squamous carcinoma or adenocarcinoma of the distal rectum, but the risk for such neoplasia seems quite low. In these cases, imaging studies do not substantially help in early detection. Therefore, although no increase in the incidence of cancer has been demonstrated in patients with chronic PCD treated with anti-tumour necrosis factor (anti-TNF) drugs, careful inspection is recommended, and anal biopsies under anaesthesia may even be needed before starting treatment with this type of drug [26].

## Determination of activity

Several PCD activity measures are used in the clinical setting; the most well known is the perianal disease activity index. It is based on the evaluation of secretion, pain, restriction of sexual activity, type of perianal fistula, and degree of induration [27].

A simpler method is fistula drainage assessment [28]. Gentle compression of the fistula path allows for characterisation of the fistula as open or closed. A fistula is considered open when content comes out upon compression, while a closed fistula is characterised by the absence of content (although it is better to describe closed fistulas as those “without drainage”). Using this method, treated patients can be classified as responders (when the drainage ceases), in remission (when the drainage decreases by >50%), or nonresponders.

In the daily clinical setting, the use of these methods in the physical examination is generally adequate. However, pelvic MRI is increasingly requested both in the initial evaluation and during follow-up [12], and whenever studies are performed for monitoring treatment, it should be mandatory [11].

## Role of MRI in follow-up

**Fig. 8:**
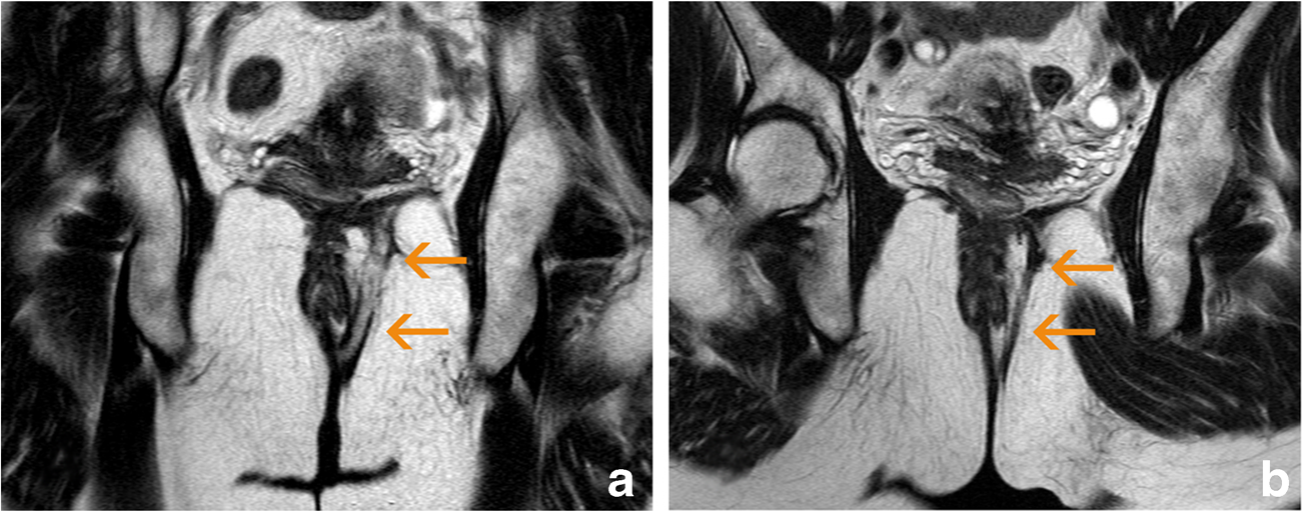
Complete response after anti-TNF therapy. **a** Axial T2-weighted sequences at baseline show a complex left trans-sphincteric fistula with hyperintense paths (arrow); **b** Axial T2-weighted sequences after 24 weeks of treatment show that all paths have decreased in thickness and exhibit hypointensity (arrow)

**Fig. 9:**
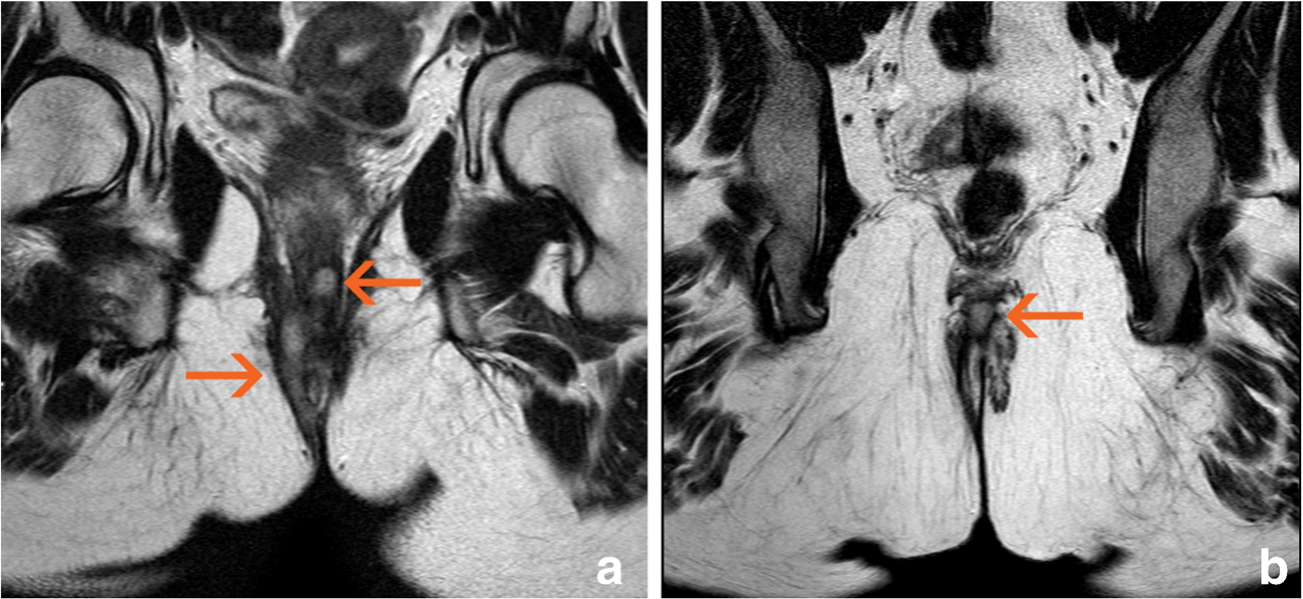
Partial response after anti-TNF therapy. **a** Axial T2-weighted sequences at baseline show a complex bilateral trans-sphincteric fistula with posterior “horseshoe” fluid. Hyperintense paths are present both near the external opening and at the intersphincteric level (arrows). **b** Axial T2-weighted sequences after 24 weeks of treatment reveal that the distal part of the fistula shows signs of healing (hypointensity); however, the intersphincteric fluid collection persists (arrow)

MRI studies have shown that closure of the external opening does not always indicate that the fistula is fully healed because signs of inflammation can persist in the internal tissues of the fistula [29] (Figs. 8 and 9). This can also be demonstrated with US [30] and indicates the importance of evaluating perianal fistulas using imaging methods not only for surgical planning but also for treatment monitoring. When using anti-TNF drugs, rigorous patient monitoring is needed not only because such drugs are expensive but also because they are not free of side effects such as infection, hypersensitivity, and others.

One of the first studies of the use of MRI for monitoring patients undergoing treatment with anti-TNF drugs was performed by Van Assche et al. [31]. They also assessed the use of a numerical scale of severity and extension based on morphological findings and the presence of signs of inflammation. The scale was used to evaluate the number and complexity of fistulas, the degree of hyperintensity in T2-weighted sequences, the presence of abscesses, and the presence of signs of rectal inflammation (Table 2). However, although the resultant score (Van Assche score) provides a quick quantitative idea of the complexity and severity of PCD, its usefulness for patient monitoring remains unclear. Karmiris et al. [32], part of this same group of authors, later evaluated 59 patients treated with infliximab and found a significant decreases in their scores in the short term (*p* < 0.002) and medium term (*p* < 0.0001), but not in the long term (e.g., 1 year). In another long-term follow-up study, Ng et al. [33] evaluated 34 patients treated with anti-TNF drugs and found that the Van Assche score was not significantly different between responders and nonresponders. Likewise, in a study carried out by Savoye-Collet et al. [34], more than 20 patients were assessed at baseline and after 1 year of treatment with anti-TNF drugs. The authors found no significant variations in the Van Assche score among responders, patients in remission, and nonresponders. Finally, Hortshuis et al. [35] studied 16 patients treated with infliximab and found no significant variations in the Van Assche score after treatment, even when clinical improvement or decreases in biological markers such as C-reactive protein had occurred.

**Table 2:** Magnetic resonance imaging-based score for severity of perianal Crohn’s disease as described by Van Assche et al. [31]

Number of fistula tracks	
None	0
Single, unbranched	1
Single, branched	2
Multiple	3
Location	
Extra- or intersphicteric	1
Transsphicteric	2
Suprasphinteric	3
Extension	
Infraelevatoric	1
Supraelevatoric	2
Hyperintensity on T2-weighted images	
Absent	0
Mild	4
Pronounced	8
Collections (cavities >3 mm diameter)	
Absent	0
Present	4
Rectal wall involvement	
Normal	0
Thickening	2

## New MRI tools

New high-field MRI equipment can provide better performance in the study of perianal fistulas. Publications regarding the performance of 3-T machines in the study of pelvic diseases such as gynaecological, prostatic, and rectal cancers have been extensively published. However, we found no reports comparing 3- and 1.5-T machines in the study of perianal fistulas. Despite this lack of information, it is expected that the superior signal-to-noise ratio of 3-T equipment will allow for improved spatial resolution compared with 1.5-T equipment as well as easier identification of fistulas and greater accuracy in three-dimensional reconstructions.

The apparent limitations of the conventional MRI approach to PCD led several groups of investigators to assess other MRI parameters that could be used to quantitatively evaluate disease severity and variations that reflect the treatment effects. The aforementioned study by Savoye-Collet et al. [34] proved that decreased intensity in T2-weighted sequences (*p* < 0.01) and a subjective decrease in enhancement after administration of intravenous gadolinium (*p* < 0.02) occurred in patients who exhibited a response or remission after treatment. The authors also observed that the disappearance of the post-gadolinium enhancement predicted clinical remission.

**Fig. 10:**
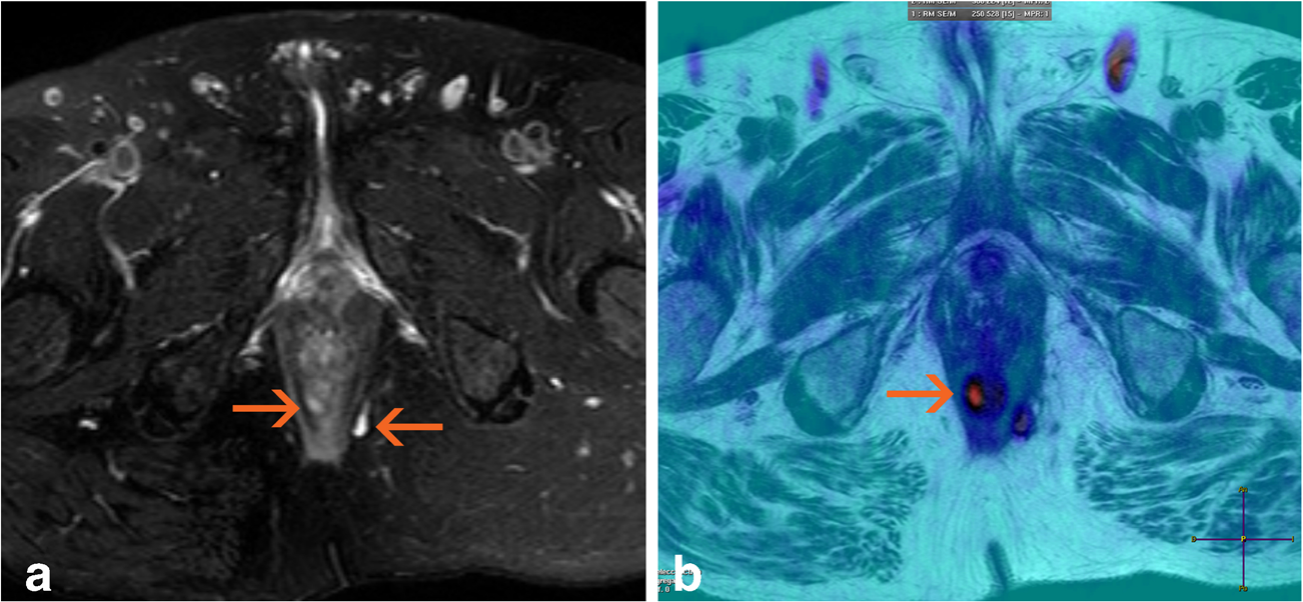
Oblique-transverse T2-weighted image (**a**) and merged view of a colour-coded map derived from an 800 s/mm^2^ b factor and a T2-weighted image (**b**). Right intersphinteric path (arrow) is clearly more conspicuous in image B

Another MRI tool is diffusion imaging. This type of sequence reflects the restrictions on the free movement of water molecules in the tissues that occur due to ischaemia, increased cellularity, or the presence of macromolecules. Although such sequences have little spatial resolution, they show greater contrast between the tissues, making the lesions easier to identify. This restriction can be measured because the image we obtain has a quantifiable apparent diffusion coefficient (ADC). The inflamed tissues usually show diffusion restriction (Fig. 10); thus, these sequences seem useful for the detection of perianal fistulas [36]. Additionally, because abscesses show low ADCs, they can be detected with diffusion imaging, particularly when the use of intravenous gadolinium is contraindicated or otherwise not possible. Dohan et al. [37] found that an ADC of <1.18 μm^2^/s can be used as a reference for diagnosing perianal abscesses with a sensitivity of 100% and specificity of 90%. However, whether diffusion sequences reflect the degree of inflammatory activity in patients with fistulas that have not been complicated by abscesses remains unclear. Although Yoshizako et al. [38] found significant differences in the mean ADC between active and inactive fistulas, other researchers did not [39, 40]. It seems necessary to expand these types of studies, especially for assessing the evolution of the ADC during treatment.

**Fig. 11:**
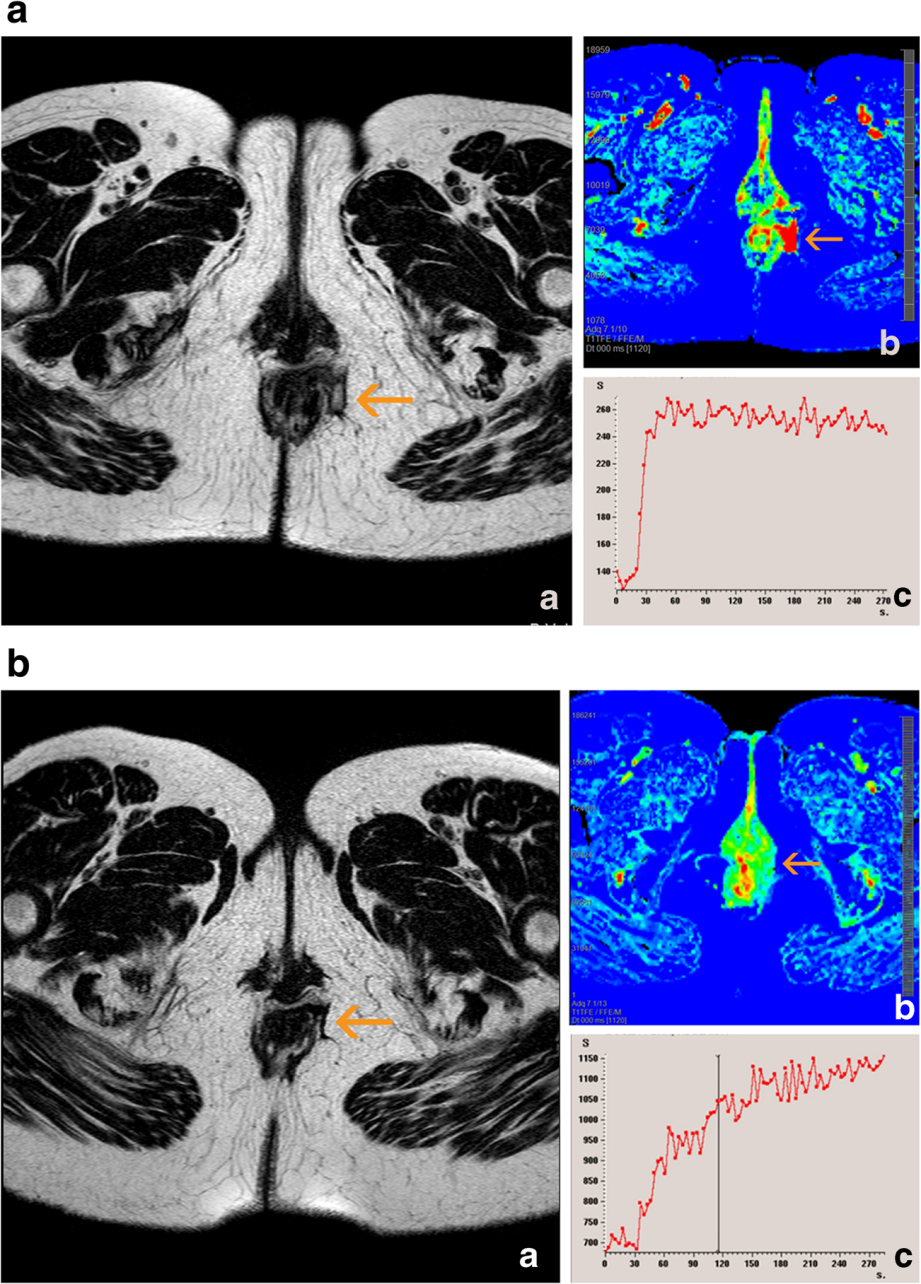
Effect of treatment on semiquantitative perfusion parameters. **a** Variation of the maximum relative enhancement at baseline. **b** Variation of the maximum relative enhancement at 4 weeks of treatment

MRI perfusion studies (dynamic contrast-enhanced MRI) reflect the degree of tissue inflammation with great fidelity. These techniques are based on the acquisition of images at the moment of passage of the contrast medium by the organ of interest, thus reflecting the dynamic response of this tissue to the arrival of blood and its subsequent distribution in the extracellular space. The analysis of signal changes as a function of time can be carried out by studying time-intensity curves from a qualitative viewpoint or using specific software that provides information on semiquantitative or quantitative parameters. The most useful semiquantitative parameters obtained from the analysis of time-intensity curves are the maximum enhancement, rate of ascent of the curve, time for the maximum value, and area under the curve (Fig 11). Quantitative parameters are based on models described by Tofts et al. [41] and include the transferability of gadolinium through the vascular endothelium (K^trans^), the fractional volume of the extracellular space (v_e_), and the relationship between these two parameters (k_ep_).

Horsthuis et al. [42] performed the first studies using these types of sequences and observed that greater numbers of pixels were rapidly enhanced in patients with more clinically severe disease, although they did not observe a correlation between the semiquantitative parameters and the perianal disease activity index. However, in a later study, Ziech et al. [43] did observe this correlation, although they only evaluated a small group of patients. Nevertheless, their study showed that 6 weeks after initiation of treatment with anti-TNF drugs, the quantitative parameter (K^trans^) had significantly decreased in treatment responders, indicating that it may be a predictive parameter of the response to treatment.

**Fig. 12:**
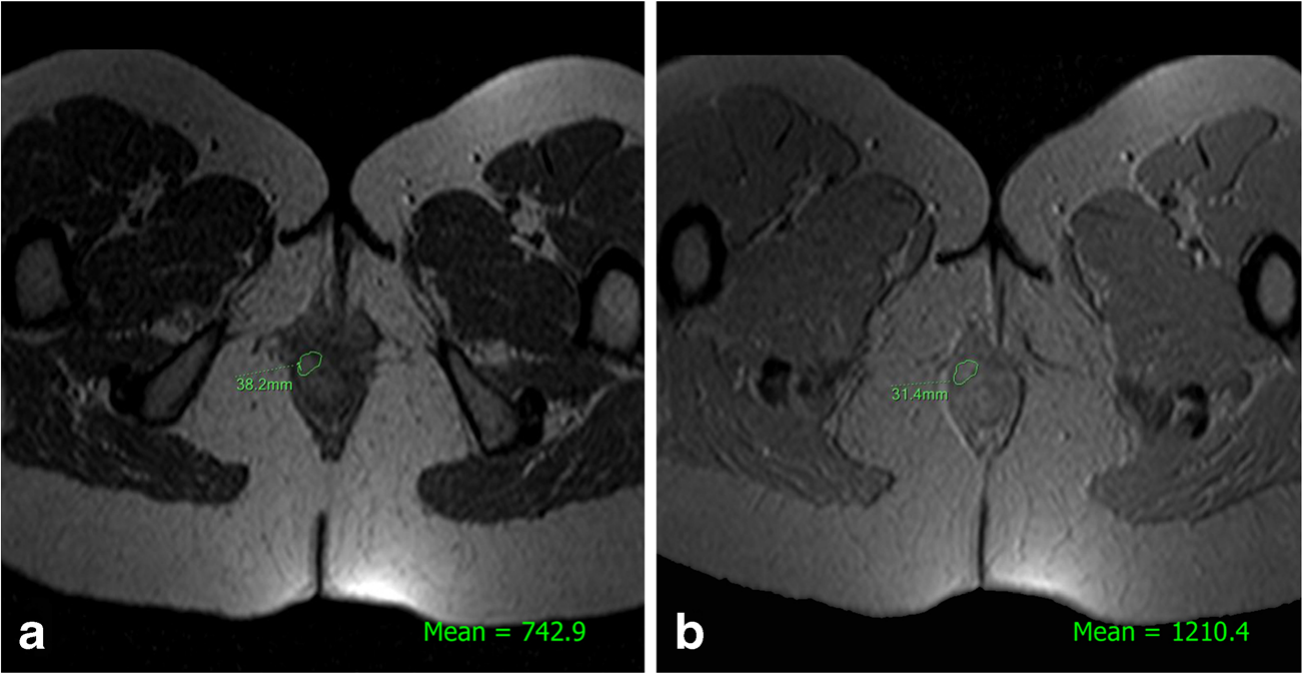
Calculation of the magnetisation transfer ratio of a fistula. **a** Signal intensity on an oblique-transverse gradient-echo image encoded at 800 off-resonance radio frequency saturation. **b** Signal intensity on an oblique-transverse gradient-echo image without radio frequency saturation. The magnetisation transfer ratio (%) is = 1- (M_sat_ image / M_nonsat_ image) × 100

Finally, in the field of non-routine MRI sequences, the most recent contribution to the assessment of the degree of PCD activity was the use of magnetisation transfer sequences. By varying the phase-coding frequencies applied, the signal is altered in accordance with the amount of macromolecules present in the tissue being studied (Fig. 12). Pinson et al. [44] found that in a group of 29 patients with CPE, the mean values of relative magnetisation transfer in the non-active group were significantly higher than those in the active group (*p* < 0.02); additionally, the values were correlated with those of the Van Assche scale (*p* < 0.05).

## Timing of MRI examination

In patients undergoing medical treatment for perianal fistulas, the clinical response is usually detected before complete disappearance of the MRI abnormalities. Several studies have shown that during the first weeks of treatment, changes occur that result in lower scores; however, it is only in the medium term (26–52 weeks) that this improvement is observed or even increased in treatment responders [32]. Likewise, another study showed that patients who were in remission at 6 months continued to be in remission 1 year later despite the fact that they had abandoned treatment [33].

In summary, it seems appropriate to propose a baseline pelvic MRI study before starting treatment and to repeat the MRI study 6 months later to assess the response. The result will be of prognostic value for the long-term disease evolution. If complete healing has not occurred, the treatment should be continued.

## Conclusions

MRI is the imaging method of choice for the initial study of patients with PCD. Endoanal or transperineal US is an alternative when experienced professionals are available to perform it.

Experienced radiologists should perform MRI with an external multichannel coil whenever possible. Ideally, a structured report should describe the path of the fistula in relation to the anatomical structures of the anus, with an attempt to identify the internal orifice and the presence of abscesses, secondary tracts, or other complications. If possible, despite the known limitations of those that are available, a score should be included to quantify the severity of the disease. In addition, attention should be paid to any other perianal manifestations of CD that may be present.

MRI is required while monitoring certain treatments, such as anti-TNF drugs. In addition to the baseline study, another MRI examination should be performed at approximately 6 months to establish a prognosis if possible. The use of new MRI sequences for the early monitoring of special treatments such as anti-TNF drugs seems promising, although more extensive studies are still required.
